# Correction to: Fitness effects of mutations to SARS-CoV-2 proteins

**DOI:** 10.1093/ve/veae026

**Published:** 2024-03-26

This is a correction to: Jesse D Bloom, Richard A Neher, Fitness effects of mutations to SARS-CoV-2 proteins, *Virus Evolution*, Volume 9, Issue 2, 2023, vead055, https://doi.org/10.1093/ve/vead055.

In the originally published version of this manuscript, several negative signs on the x and y axes in Figure 3 were erroneously rendered as the number 6.

These errors have been corrected.

